# Delayed abdominal wall abscess after abdomino-perineal resection simulating local recurrence of rectal cancer

**DOI:** 10.1186/2193-1801-3-681

**Published:** 2014-11-20

**Authors:** Kazushige Kawai, Eiji Sunami, Takeshi Nishikawa, Junichiro Tanaka, Toshiaki Tanaka, Tomomichi Kiyomatsu, Keisuke Hata, Hiroaki Nozawa, Shinsuke Kazama, Soichiro Ishihara, Hironori Yamaguchi, Joji Kitayama, Toshiaki Watanabe

**Affiliations:** Department of Surgical Oncology, Faculty of Medicine, the University of Tokyo, 7-3-1 Hongo, Bunkyo-ku, Tokyo, 113-0033 Japan

**Keywords:** Abdominal wall abscess, Rectal cancer, Recurrence, Abdominoperineal resection

## Abstract

**Introduction:**

We report a rare case of delayed abdominal wall abscess after abdominoperineal resection (APR) for rectal cancer.

**Case description:**

A 63-year-old woman was diagnosed with rectal cancer and received chemo-radiotherapy, followed by APR. One year after surgery, the patient complained of pain and skin redness in the lower abdomen. A low-density mass lesion with 5.9-cm diameter was found in the lower abdominal wall by computed tomography, which showed high uptake on positron-emission tomography. These findings suggested the possibilities of either delayed abscess formation or abdominal wall recurrence of rectal cancer with central necrosis. Percutaneous drainage was performed. The content was a purulent exudate, without neoplastic cells in the cytology. The lesion quickly disappeared after the drainage, and no recurrence of the tumor was observed for more than 2 years.

**Discussion and evaluation:**

In this case, the un-absorbable yarn, such as silk, has not been used during the operation, no foreign body was retained in the abdominal wall, and there was no associated inflammatory bowel disease. Use of neoadjuvant chemoradiotherapy was the only possible cause of delayed abscess formation in this case.

**Conclusion:**

In case local recurrence is suspected by imaging modalities in the postoperative of colorectal cancer, especially those with precedent chemoradiotherapy or radiotherapy, although rare, the possibility of a delayed abscess formation should also be considered.

## Introduction

Surgical site infection (SSI) is a common complication of gastrointestinal surgery, especially colorectal surgery. Although rectal surgery is accompanied by a higher incidence of SSI compared to colon surgery (17–28%) (Awwad et al. 2010; Bullard et al. 2005), most SSIs develop within a few weeks after surgery, rarely being found months after surgery. Here, we describe a case of an abdominal wall abscess diagnosed 1 year after the abdominoperineal resection (APR) for rectal cancer, which was difficult to distinguish from the recurrence of cancer with imaging modalities.

## Case report

**Figure 1:**
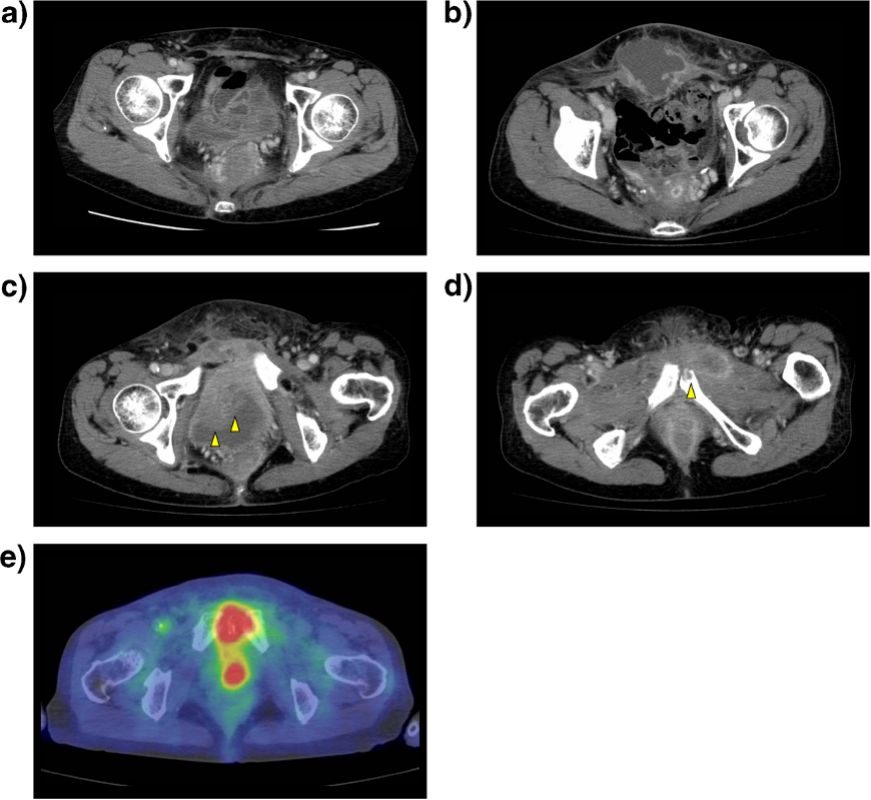
**Postoperative imaging modalities. a)** Computed tomography (CT) 6 months after surgery. No abscess is seen. **b-d)** CT 1 year after surgery. A marginally enhanced low-density mass lesion is seen in the lower abdominal wall **(b)**. The mass lesion is adjacent to the bladder wall with bladder wall thickness (**c**, yellow arrow) and the pubic bone with partial bone destruction (**d**, yellow arrow). The tumor shows high uptake on positron-emission tomography **(e)**.

A 63-year-old woman was referred to our hospital with the diagnosis of rectal cancer. The cancer was located 2 cm from the anal verge; the clinical depth of the cancer was T4, and no lymph node or distant metastasis was evident on computed tomography (CT). Preoperative chemoradiotherapy was performed with a total dose of 50.4 Gy of radiation and concomitant oral administration of tegafur-uracil, followed by APR with curative intent. The pathological finding of the resected specimen was ypT2N0M0 stage I. The postoperative course was uneventful without any SSI. No adjuvant chemotherapy was administered, and no recurrence or abscess formation was seen on the CT imaging 6 months after the APR (Figure 1a).

One year after the APR, the patient complained of pain and redness of the skin in the lower abdomen. The CT imaging showed a 5.9-cm diameter low-density mass lesion in the lower abdominal wall (Figure 1b). The mass lesion, adjacent to the bladder and to the pubic bone, was marginally enhanced, and thickness of the bladder wall (Figure 1c) and partial destruction of the pubic bone (Figure 1d) were observed. The positron-emission tomography (PET) revealed high uptake of fluorodeoxyglucose by the tumor, with a maximum standardized uptake value of 11.1 (Figure 1e). The laboratory data was indicative of acute inflammation (white blood cell count, 11800/μL; C-reactive protein, 18.1 mg/dL), without elevation of tumor markers (carcinoembryonic antigen, 2.4 ng/mL; CA19-9, 10 U/mL). From these findings, we suspected of either a delayed abscess formation or the recurrence of rectal cancer in the abdominal wall with central necrosis; hence, percutaneous drainage was performed for therapeutic and diagnostic purposes. A purulent exudate was removed from the tumor, and the bacteriological culture revealed the growth of group G *Streptococcus.* The cytological examination of the exudate was negative for neoplastic cells. After the drainage of the abscess, followed by antibiotic administration, the tumor quickly regressed, without recurrence of cancer or the abscess even 2 years after the treatment.

## Discussion

Delayed abscess formation in the abdominal wall is a rare postoperative complication, and as late as the abscess develops, the more difficult the differential diagnosis. Surgical treatment of infectious diseases, such as cholecystitis or appendicitis, is known to be associated with a higher risk of late abscess formation, due to retained stones in the abdominal wall at the time of surgery (Calkins et al. 2007; Degrate et al. 2011; Imamoglu et al. 2004). Moreover, mesh plug repair of an incisional or inguinal hernia, as well as the use of silk yarn in surgical suture, are associated with late abscess formation (Lapus and Baker 2010; Maluf et al. 2007; Pandey et al. 2010). Therefore, retained foreign bodies, such as stones, use of artificial mesh, or unabsorbable surgical suture may be important risk factors for delayed abscess formation. Radiotherapy, which may cause necrosis of the irradiated tissue, as a rare late complication, is another cause of delayed infection. Necrosis of the bladder diagnosed 45 years after radiotherapy (Rantala et al. 2009) and necrotizing fasciitis nine months after chemoradiotherapy for head and neck cancer (Serra-Aracil et al. 2011) have been reported.

The incidence of SSI is higher after APR compared to other rectal surgeries (Simopoulos et al. 2000), and in our institution, 38% of the 150 patients who received APR between 2000 and 2012 developed SSI. However, reports concerning delayed abscess formation associated with APR are limited. In 1978, Smith et al. reported seven cases of late occurrence of perineal wound abscess years after total colectomy, six of which were treated with proctocolectomy because of ulcerative colitis (Smith et al. 1978). They suggested the association between inflammatory bowel disease or concomitant steroid administration and delayed perineal wound infection.

Group G streptococcus was responsible for the infection of the present case. Group G streptococcus is classified as beta-haemolytic streptococcus. Because group G streptococcus is part of the normal commensal flora of the human upper airway and skin, the origin of the abscess should not be bowel penetration or fistula, but bacteria derived from the skin at the time of surgery, which remained occult for a long time after the operation. The incidence of group G streptococci-related bacteraemia in adult patients is reported to be increasing in recent years (Zuvela et al. 2012).

In the present case, the differential diagnosis was difficult, especially with recurrent tumor, because the abnormal thickness of the bladder wall and the bone destruction observed in the CT scan, and the high tumor intake in PET, in addition to the long time course after the operation (1 year), were strongly suggestive of locally recurrent cancer. In this case, the un-absorbable yarn, such as silk, has not been used during the operation, no foreign body was retained in the abdominal wall, and there was no associated inflammatory bowel disease. Use of neoadjuvant chemoradiotherapy was the only possible cause of delayed abscess formation in this case.

In conclusion, although rare, the possibility of delayed abscess formation should be considered as a differential diagnosis in case of suspected local recurrence by imaging modalities in the late postoperative of colorectal cancer cases with precedent chemoradiotherapy or radiotherapy.

### Approval by an ethics committee

An approval by an ethics committee was not applicable.

### Informed consent

Informed consent was obtained from the patient for being included in the study.
